# Reference *in-vitro* dataset for inertial-sensor-to-bone alignment applied to the tibiofemoral joint

**DOI:** 10.1038/s41597-021-00995-8

**Published:** 2021-08-05

**Authors:** Ive Weygers, Manon Kok, Thomas Seel, Darshan Shah, Orçun Taylan, Lennart Scheys, Hans Hallez, Kurt Claeys

**Affiliations:** 1 KU Leuven campus Bruges, Department of Rehabilitation Sciences, Bruges, 8200 Belgium; 2 TU Delft, Department of Mechanical, Maritime and Materials Engineering, Delft, 2628 CD the Netherlands; 3 Friedrich-Alexander-Universität Erlangen-Nürnberg, Department Artificial Intelligence in Biomedical Engineering, Erlangen, 91054 Germany; 4 KU Leuven, Department of Development and Regeneration, Institute for Orthopaedic Research and Training (IORT), Leuven, 3000 Belgium; 5 University Hospitals Leuven, Division of Orthopaedics, Leuven, 3000 Belgium; 6 KU Leuven campus Bruges, Department of Computer Sciences, Bruges, 8200 Belgium

**Keywords:** Mathematics and computing, Medical research, Data processing

## Abstract

Skin-attached inertial sensors are increasingly used for kinematic analysis. However, their ability to measure outside-lab can only be exploited after correctly aligning the sensor axes with the underlying anatomical axes. Emerging model-based inertial-sensor-to-bone alignment methods relate inertial measurements with a model of the joint to overcome calibration movements and sensor placement assumptions. It is unclear how good such alignment methods can identify the anatomical axes. Any misalignment results in kinematic cross-talk errors, which makes model validation and the interpretation of the resulting kinematics measurements challenging. This study provides an anatomically correct ground-truth reference dataset from dynamic motions on a cadaver. In contrast with existing references, this enables a true model evaluation that overcomes influences from soft-tissue artifacts, orientation and manual palpation errors. This dataset comprises extensive dynamic movements that are recorded with multimodal measurements including trajectories of optical and virtual (via computed tomography) anatomical markers, reference kinematics, inertial measurements, transformation matrices and visualization tools. The dataset can be used either as a ground-truth reference or to advance research in inertial-sensor-to-bone-alignment.

## Abstract (summary)

**Table:** 

Measurement(s)	marker trajectory • anatomical landmark • Inertia
Technology Type(s)	optical motion capture system • computed tomography • inertial measurement device
Factor Type(s)	movement plane • movement duration • movement excitation • tibiofemoral flexion range of motion
Sample Characteristic - Organism	Homo sapiens
Sample Characteristic - Environment	cadaver

Machine-accessible metadata file describing the reported data: 10.6084/m9.figshare.14987013

## Background & Summary

In recent decades, researchers relied on laboratory equipment and computational methods to track human movements^1^. Optical motion capture (OMC) is often used to track body movements via skin-attached reflective markers and infrared cameras^2^. However, an OMC is limited in physical space and difficult to apply in outside-lab environments^2^, e.g., to measure early postoperative adaptations in a hospital^3^. Skin-attached inertial measurement units (IMUs) provide an alternative that can be applied in these demanding environments^2^. However, their noisy and biased measurements make the inference of kinematics a complex and highly studied sensor fusion problem^4–6^ that furthermore requires a sufficient background in the field of biomechanics.

While interest in inertial sensors is rising, it remains an open question how good inertial-sensor-to-bone alignment methods relate the sensor’s axes with the underlying anatomical axes^7^ as defined by the clinical definitions^8,9^. Only after an accurate alignment, comparable kinematic measures can be obtained. The vast majority of IMU-based kinematic studies assume that the skin-attached IMUs’ sensing axes approximately align with the underlying anatomical segmental axes^10,11^. Naturally, violations against such assumptions yield kinematic cross-talk errors (where parts of the rotations on certain axes are sensed on other axes)^12,13^, which makes interpretation notoriously difficult. Other approaches define functional movements or poses to conduct the sensor-to-bone alignment, but their accuracy highly depends on the ability of the subject or instructor to execute movements around isolated axes. More promising are model-based inertial-sensor-to-bone alignment methods that aim to relate inertial measurements with a model of the joint’s mechanics, while overcoming the need for calibration movements and sensor placement assumptions^14–17^. Such methods require a sufficient amount of movement, for the model to become manifest in the inertial measurements. However, it is not known how well these model-based alignment methods are able to identify the direction of underlying joint axes that relate with anatomical landmarks, as defined by the clinical definitions^8,9^. Furthermore, it is not clear which movements are necessary in order to construct the alignment. Hull^12^ highlighted that extensive validation of these alignment methods is often underestimated and most often done by means of their resulting joint kinematics, with respect to an OMC kinematic reference^14,18–20^. It is thus not straightforward to evaluate alignment models when errors from inertial sensor orientation estimation and kinematic cross-talk due to mis-alignments are intertwined^12^ and possibly disturbed by skin motion artifacts, which led researchers to question an OMC as an appropriate reference^12,13,21^.

**Fig. 1:**
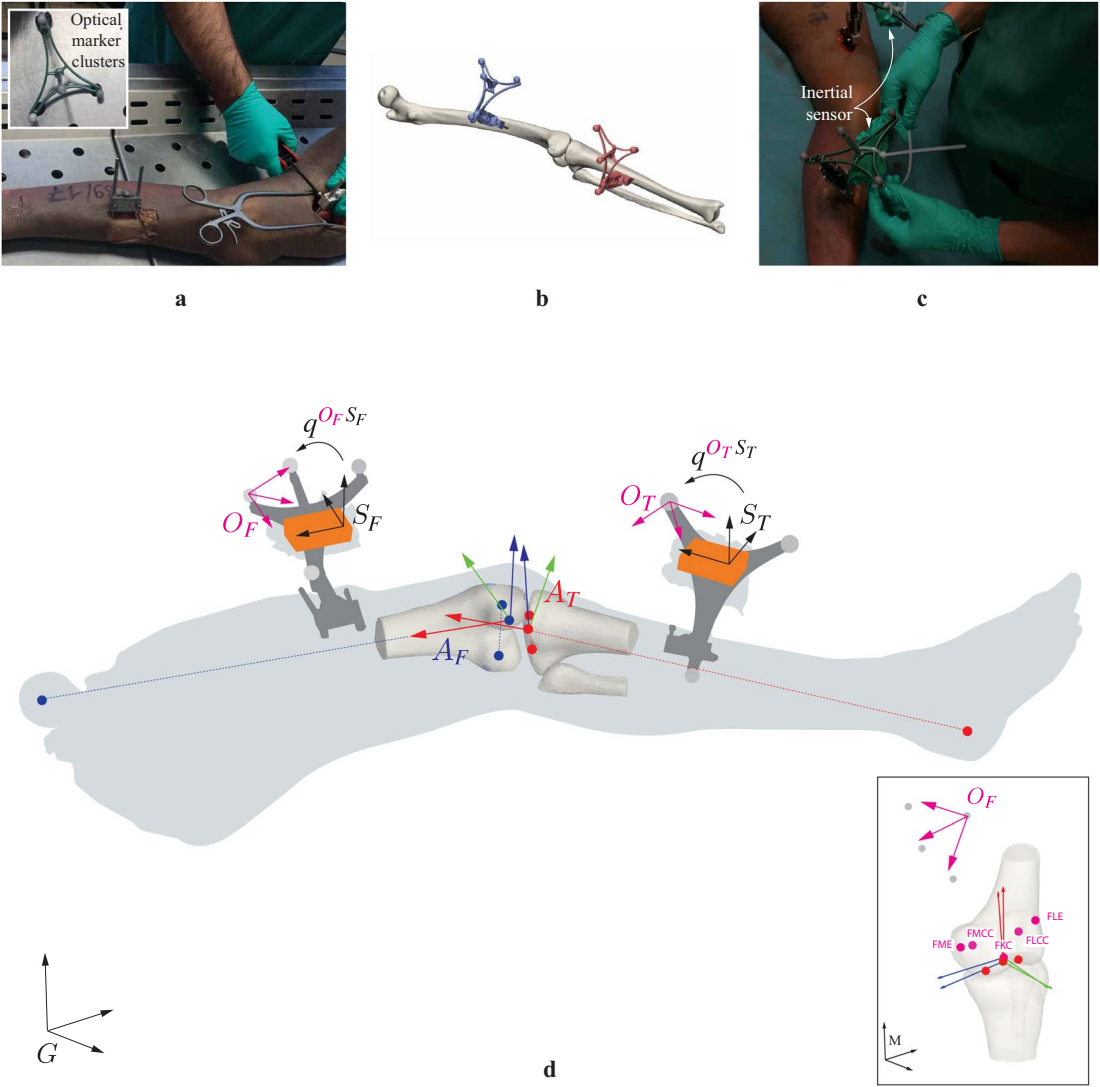
Experimental set-up. (**a**) A cadaveric lower limb is equipped with rigidly attached bone-pins, at the medial side of the femur (_*F*_) and tibia (_*T*_) segments. Each bone-pin is equipped with retro reflective marker clusters (that are used to create optical marker-based coordinate systems *O*_*F*_ and *O*_*T*_) and inertial sensors (orange boxes) with sensor coordinate systems *S*_*F*_ and *S*_*T*_. (**b**) Three-dimensional surface bone models are reconstructed for the femur and tibia bone and osseous anatomical landmarks are identified within Mimics. Anatomical reference coordinate systems *A*_*F*_ and *A*_*T*_ are defined on the base of virtual anatomical landmarks. Anatomical landmarks are furthermore rotated into a common intermediate coordinate system (pink) within the CT-scan coordinate system *M*, to rotate the landmarks into the optical motion capture reference frame *G*. The full explanation of all abbreviations of the annotated anatomical landmarks can be found in Table 1. (**c**) Inertial sensor are rigidly attached on the femur and tibia-attached bone-pins via zip ties. The alignment rotations ${q}^{{O}_{F}{S}_{F}}$ and ${q}^{{O}_{T}{S}_{T}}$ define the rotation from coordinate frame *S* to coordinate frame *O* for the femur and tibia-attached inertial sensors. As a result, all coordinate systems can be tracked with respect to the optical motion capture reference coordinate system *G*, after the necessary coordinate system transformations. (**d**) Illustration of the measurement set-up with the different coordinate frames.

Previous datasets that combine different modalities, including inertial sensor measurements, have been published for the assessment of human grasping^22,23^ and for the recognition of gait adaptations^24^. To our knowledge, the proposed dataset is the first publicly accessible dataset that provides an anatomical reference for inertial-sensor-to-bone alignment methods. We focus on the tibiofemoral (TF) joint that is most studied for inertial-sensor-to-bone alignment^7^ and report a rich dataset of dynamic movements on a cadaver that were recorded with multi-modal measurements including trajectories of optical markers and virtual (through volumetric computed tomography (CT) scanning) anatomical markers, reference joint kinematics and inertial measurements (Fig. 1d). Within the measurement protocol, regular static measurements for gyroscope bias estimation and compensation^25^ and slow rich movements for magnetometer calibration^26^ are included. This work provides the methodological details to allow for replication of the developed validation strategy. The necessary alignment matrices are provided to validate IMU-based estimates of underlying anatomical axes, and compare estimates in the underlying anatomical coordinate systems. The measurement protocol intrinsically overcomes the ethical difficulties for an *in vivo* measurement protocol^27^ and can aid in a better understanding and advancement on inertial sensor-based biomechanical modeling^14,15^ of the complex tibiofemoral joint^28^. The current dataset can furthermore be used for the validation of inertial-sensor-based identification of biomechanical parameters, e.g, joint center position^29,30^and is expected to be used repeatedly as a ground-truth reference in the multidisciplinary field that links sensor fusion and biomechanics.

## Methods

### Specimen overview

A complete fresh frozen cadaveric lower limb, disarticulated at the level of the hip was used for the experiment. The female specimen (age: 52, left leg) did not show any history in knee injuries, e.g., meniscal lesions, ligament ruptures or knee osteoarthritis, and was obtained from the licensed Institute for Orthopaedic Research and Training (IORT, Leuven, Belgium). The use of human specimen and all test procedures were approved by the local ethical committee UZ Leuven and registered at the Belgian National Council for Bioethics (number: NH019) prior to experimental testing.

### Experimental work-flow

The specimen was kept in a freezer and removed twenty-four hours prior to experimentation, to allow sufficient time for thawing. First, the specimen was equipped with clusters of spherical infrared reflective markers that were rigidly attached via bone-pins at the medial side, mid-distance onto the femur and tibia segments as illustrated in Fig. 1a. A minimum of three non-collinear markers were necessary to establish a coordinate system, but four markers per cluster were used to reduce registration errors from occlusion in the optical motion tracking system. Second, a volumetric computed tomography scan (Siemens Somatom Force, Siemens Healthcare, Erlangen, Germany) was obtained from the frozen specimen, after placement of the bone-pins. Images were obtained with a slice thickness of 0.6 mm. The computed tomography scans were analyzed with Mimics (Materialise, Haasrode, Leuven, Belgium) to create three-dimensional (3D) reconstructions of both femur and tibia bones (Fig. 1b). Afterwards, the necessary anthropometric osseous anatomical landmarks were identified to construct joint coordinate systems for the femur and tibia from the 3D surface bone models, following Grood and Suntay^8^. The marker clusters were localized in both the CT-scan images and the optical motion capture system. This aids in the spatial alignment between the two reference systems and the registration of virtual anatomical landmarks. Before conduction of dynamic experiments, each bone-pin was equipped with a rigidly attached wireless inertial sensor (Mtw Awinda, Xsens, Enschede, the Netherlands) via zip ties (Fig. 1c). A hardware time synchronization was used to simultaneously capture optical marker trajectories by a six-camera OMC (MX + , Vicon, Oxford, UK) and inertial measurements, both with a sample rate of 100 Hz.

### Measurement protocol

Data of multiple dynamic experiments were collected by experienced physiotherapists. Prior to each trial, a pseudo-static time-period was introduced where the specimen was held still for approximately five seconds in the position described by the measurement protocol. For each trial, the specimen was then moved in an unloaded position by hand from full extension to a desired level of tibiofemoral flexion, following a predefined measurement protocol by altering the following protocol variables:

1. *Movement plane* – We differentiated between movements in a fixed vertical movement plane (horizontal femoral-fixed flexion-axis), fixed horizontal movement plane (vertical femoral-fixed flexion-axis) and a mixed movement plane that could change its orientation over time. This overcomes a fixed horizontal axis-setup on a mechanical knee rig^31^ that may prevent identification of axis direction, (i.e., a problem of sign pairing may arise such that a femur-fixed flexion-axis that is pointing in medial direction, is estimated to point in lateral direction, but with the same orientation^32,33^).
2. *Movement duration* – 15 seconds, 30 seconds or 120 seconds, to allow for both quick processing as well as the introduction of drift-effects^4,34^.
3. *Movement excitation* – We instructed different movement excitation levels as slow, fast and mixed, and later quantified it as slow (norm angular velocity 0.85 ± 0.63 rad/s (femur-attached inertial sensor) and 0.72 ± 0.60 rad/s (tibia-attached inertial sensor)), fast (norm angular velocity 1.63 ± 1.05 rad/s (femur-attached inertial sensor) 1.60 ± 1.20 rad/s (tibia-attached inertial sensor)) and mixed (a random sequence of slow and fast movement periods) to mimic a wide range of movement dynamics.
4. *Tibiofemoral flexion range of motion (RoM)* – We differentiated between tibiofemoral flexion RoM of 60 degrees, in line with expected RoM during normal gait and 110 degrees to simulate functional squat movements^13^.

The measurement protocol included every possible combination of these four protocol variables and a custom script gave real-time feedback on the RoM to guide the physiotherapists in actuating the specimen. Experiments were executed with care to ensure that the limb was supported in the same way for all runs. Additionally, functional limb poses and movements were recorded and are described as:

- *A vertically positioned* specimen (horizontal femur-fixed flexion-axis) with a manually fixated tibia at the ankle joint or femur at the femoral head. Followed by a set of manually induced rotations of the femur or tibia from full extension up to maximal tibiofemoral flexion.
- *A vertically positioned* specimen (horizontal femur-fixed flexion-axis) with a manually fixated femur at the femoral head. Followed by a set of isolated manually induced tibia internal and external rotation movements within the maximum physical range of motion.
- *A horizontally positioned* specimen (vertical femur-fixed flexion-axis) with a manually fixated tibia at the ankle joint or femur at the femoral head. Followed by a set of manual induced rotations of the femur or tibia from full extension up to maximal tibiofemoral flexion.

Although not in line with the intuition of model-based alignment methods that aim to be independent from calibration movements. These additional functional movements enrich the dataset with a debugging purpose on simple functional limb motions.

### Spatial alignment

We differentiate between the following Cartesian coordinate systems in which measurements can be expressed: 1) the global reference coordinate system *M*, in which the anatomical landmarks from the 3D surface bone models are defined, 2) the global reference coordinate system *G* of the OMC in which marker trajectories are expressed, 3) the sensor coordinate system *S* in which the inertial measurements and estimated biomechanical parameters are expressed, 4) the navigation coordinate system *N* that serves as a reference for the sensor orientation *q*^*NS*^. Since the optical markers on femur and tibia are identified in both the CT-scan (*M*) and in the optical motion capture system (*G*), a common intermediate coordinate system *O* can be defined on the basis of three non-collinear optical markers *O*1, *O*2 and *O*3 with normalized base vectors; $x=O1\to O2$, $z=(O1\to O3)\times (O1\to O2)$, $y=x\times z$, which was made right-handed, by inverting *z* if $x\times y\ne z$. This allows us to describe virtual anatomical marker trajectories within *G* after the necessary rotations from reference coordinate frame *M* to reference coordinate frame *G* via intermediate coordinate frame *O*.

Furthermore, the sensor’s internal on-chip sensing axes are not perfectly aligned with the IMU-case, nor with a coordinate system on the basis of three surrounding rigidly attached optical markers *O*_*F*_, *O*_*T*_. A constant misalignment that describes the rotation from inertial sensor coordinate system to the optical marker-based coordinate system was identified for each sensor (${q}^{{O}_{F}{S}_{F}}$, ${q}^{{O}_{T}{S}_{T}}$) with the closed-form solution in Theorem 4.1 from J. D. Hol^25^ by using measured (from the inertial measurements) and approximated (from the optical cluster markers) angular velocities as an input^25^, from all experimental data points (excluding the pseudo-static time-period) of all trials, to cover most of the rotation space^35^.

## Data Records

**Fig. 2:**
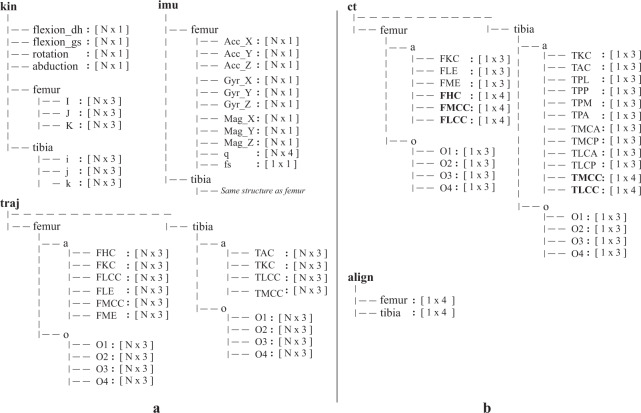
The data structure that is used for all experimental trials (**a**), and the CT-scan landmarks (**b**). The data dimensions are provided between brackets. The data in (**a**) is grouped per modality and segment with the abbreviations: (kin) reference joint kinematics, (imu) inertial measurements, (traj.o) optical and (traj.a) virtual anatomical marker trajectories. For the CT-scan landmark positions in (**b**) a similar grouping is used. Anatomical landmarks in bold represent spheres and circles. The first three coordinates define the coordinates of the center and a fourth coordinate was used for the radius where appropriate. N denotes the amount of samples. An explanation of each individual abbreviation in the data structure can be found in Table 1 for the structure in (**a**) and in Table 2 for the structure in (**b**).

The data records and a dataset summary spreadsheet (*Data_Summary.xlsx*) are available through the Figshare repository^36^ (10.6084/m9.figshare.c.5328773). The dataset summary spreadsheet provides additional information for each trial including the measurement protocol variables, file-size and the amount of recorded samples (including the pseudo-static period at the start of each trial). Raw and derived data from different modalities (optical marker trajectories, inertial measurements, reference kinematics, alignment matrices) were structured into separate.mat datafiles (structure arrays data-type) per trial with a custom Matlab (R2019b, Mathworks, Natick, USA) script. Each datafile has the following naming convention *“MovementPlane”_“Duration”_“Excitation”_“RoM”* and is structured as illustrated in Fig. 2a. The naming convention for the functional movements is provided in the dataset summary spreadsheet. Table 1 provides a detailed explanation on the abbreviations used in the data structure, including the unit and the reference coordinate system in which the data are expressed. The following sections further describe the raw and derived data that are available within each datafile.

**Table 1:** Abbreviations used in the datafile structures of the experimental trials (Fig. 2a) together with a full explanation, the unit and the reference coordinate system in which the measures are expressed.

Data structure abbreviation	Explanation	Unit	Reference coordinate system
flexion_dh	Tibiofemoral flexion (following Dabirrahmani and Hogg)^40^	[deg]	N/A
flexion_gs	Tibiofemoral flexion (following Grood and Suntay)^8^	[deg]	N/A
rotation	Tibia external rotation^8^	[deg]	N/A
abduction	Tibiofemoral abduction^8^	[deg]	N/A
I|J|K	Base vectors for the femoral Cartesian coordinate system^8^	unit vector	G
i|j|k	Base vectors for the tibial Cartesian coordinate system^8^	unit vector	G
Acc_X|Y|Z	Accelerometer measurements on the X, Y, Z sensor axes	[m/s^2^]	S
Gyr_X|Y|Z	Gyroscope measurements on the X, Y, Z sensor axes	[rad/s]	S
Mag_X|Y|Z	Magnetic field strength measured on the X, Y, Z sensor axes	[a.u.]^37^	S
q	Sensor orientation estimate *q*^*NS*^ expressed in terms of a unit quaternion	unit vector	N/A
fs	Sample frequency	[Hz]	N/A
FHC	Femoral Hip Center position	[mm]	G
FKC	Femoral Knee Center position	[mm]	G
FLCC	Femoral Lateral Condyle Center position	[mm]	G
FLE	Femoral Lateral Epicondyle position	[mm]	G
FMCC	Femoral Medial Condyle Center position	[mm]	G
FME	Femoral Medial Epicondyle position	[mm]	G
TAC	Tibial Ankle Center position	[mm]	G
TKC	Tibial Knee Center position	[mm]	G
TLCC	Tibial Lateral Condyle Center position	[mm]	G
TMCC	Tibial Medial Condyle Center position	[mm]	G
O1-O4	Optical marker position	[mm]	G

### Raw data

#### 3D surface bone models

The surface bone models of both femur (tibia.stl) and tibia (femur.stl) segments provide additional insight and allow for the identification of other custom landmarks. We also provided a reduced vertex version of both surface bone models (indicated by’_red’ suffix) that can be used for rapid plotting. From these models, anatomical landmarks and optical markers were identified on the 3D surface bone models and structured in (ct.mat) as depicted in Fig. 2b. Table 2 provides a full explanation of the identified points, spheres and circles. Note that coordinates are expressed in the reference coordinate system of the CT-image.

**Table 2:** Abbreviations used in the datafile structure for the computed tomography scan (Fig. 2b) together with the type (point, circle or sphere) a full explanation, the unit and the reference coordinate system in which the measures are expressed.

Data structure abbreviation	Type	Explanation	Unit	Reference coordinate system
FKC	point	Femoral Knee Center	[mm]	M
FLE	point	Femoral Lateral Epicondyle	[mm]	M
FME	point	Femoral Medial Epicondyle	[mm]	M
TKC	point	Tibia Knee Center	[mm]	M
TAC	point	Tibia Ankle Center	[mm]	M
TPL	point	Tibia Plateau most Lateral point	[mm]	M
TPP	point	Tibia Plateau most Posterior point	[mm]	M
TPM	point	Tibia Plateau most Medial point	[mm]	M
TPA	point	Tibia Plateau most Anterior point	[mm]	M
TMCA	point	Tibia Medial Plateau most Anterior point	[mm]	M
TMCP	point	Tibia Medial Plateau most Posterior point	[mm]	M
TLCA	point	Tibia Lateral Plateau most Anterior point	[mm]	M
TLCP	point	Tibia Lateral Plateau most Posterior point	[mm]	M
TMCC	circle	Tibia Medial Plateau Center	[mm]	M
TLCC	circle	Tibia Lateral Plateau Center	[mm]	M
FHC	sphere	Femur Hip Center	[mm]	M
FMCC	sphere	Femur Medial Condyle Center	[mm]	M
FLCC	sphere	Femur Lateral Condyle Center	[mm]	M
O1-O4	point	Optical marker position	[mm]	M

#### Optical marker trajectories

Six (MX + , Vicon) infrared cameras positioned in a half-sphere around the specimen recorded the trajectories of the optical marker clusters that were rigidly attached at the femur and tibia segments. The raw marker trajectories were processed in Vicon Nexus (Vicon, Motion Systems, Oxford, UK) using the processing pipelines for the labeling and gap filling. Gap-filling was done with a cubic spline interpolation. For each trial, the processed, unfiltered optical marker trajectories of the four markers per cluster (*O*_1_-*O*_4_) (both for femur and tibia) were included in the datafiles.

#### Inertial measurements

Each inertial sensor that was attached on the specimen consisted of a gyroscope, an accelerometer and a magnetometer that measured the sensor’s angular velocity, external specific force (comprised of the sensor’s acceleration and gravity component) and magnetic field strength, in three orthogonal directions. The sample rate *f*_*s*_ of the inertial sensors and an estimate of its orientation expressed in terms of a unit quaternion ${q}_{t}^{NS}$ with respect to a sensor navigation coordinate system *N* (typically aligned with the Earth’s gravity and the local magnetic field) is provided in each datafile. The subscript _t_ explicitly denotes the time-dependency. The sensor fusion algorithm that was used to obtain these orientation estimates (Xsens Kalman filter) is proprietary of the sensor^37^, but any custom or available^38,39^ orientation estimation strategy can be applied to the available raw inertial measurements. Also, an accurate orientation of the sensor can be obtained from the available marker trajectories after the necessary spatial alignment^25^.

Additionally, regular measurements for gyroscope bias estimation and magnetometer calibration were included and annotated in the dataset summary spreadsheet. The gyroscope bias can be estimated from measurements where the sensor-equipped specimen was kept stationary for approximately ten seconds^4^. If magnetometer readings are a desired input of the inertial-sensor-based alignment algorithm subject to validation, possible magnetic disturbance (due to mounting of the sensor on magnetic objects or the presence of magnetic equipment in the lab) can be compensated for^26^ using these associated recordings of slow movements in all directions of the data acquisition.

### Derived data

#### Virtual anatomical marker trajectories and sensor alignment rotations

A spatial alignment was used to describe the trajectory of virtual anatomical landmarks within the OMC reference coordinate frame *G*. Figure 2a describes the data structure used for all trials, including the virtual anatomical landmarks. Furthermore, the constant misalignments rotations ${q}^{{O}_{F}{S}_{F}}$ and ${q}^{{O}_{T}{S}_{T}}$ are provided (align.mat) for each sensor and describe the rotation from the inertial sensor coordinate frame to the optical marker-based coordinate frame.

#### Reference kinematics

Reference kinematics consisting of tibiofemoral flexion, tibia external rotation and tibiofemoral abduction were calculated from the virtual anatomical marker trajectories following the standards for reporting clinical rotations of the knee^8^ and are provided as a reference for each trial. The motions in the measurement protocol contained tibiofemoral flexion angles >90° and in hyper-extension (<0°). This would lead to clipping in the TF flexion kinematics when calculated following Grood and Suntay^8^. We therefore used the adaptation from Dabirrahmani and Hogg^40^ to provide a kinematic reference for all ranges of tibiofemoral flexion. The provided kinematics allow in-depth assessment of inertial-sensor-to-bone alignment methods by feeding the algorithm with samples that are measured during specific ranges of clinical rotations. We also provide the time-dependent base vectors for the femoral (*I*_*t*_, *J*_*t*_, *K*_*t*_) and tibial (*i*_*t*_, *j*_*t*_, *k*_*t*_) Cartesian coordinate systems as a reference. These vectors can for example be used to visualize the movement from a static tibia or femur anatomical coordinate frame perspective or to rotate estimated joint axes into anatomical coordinate systems for validation purposes.

#### Visualization tools

**Fig. 3:**
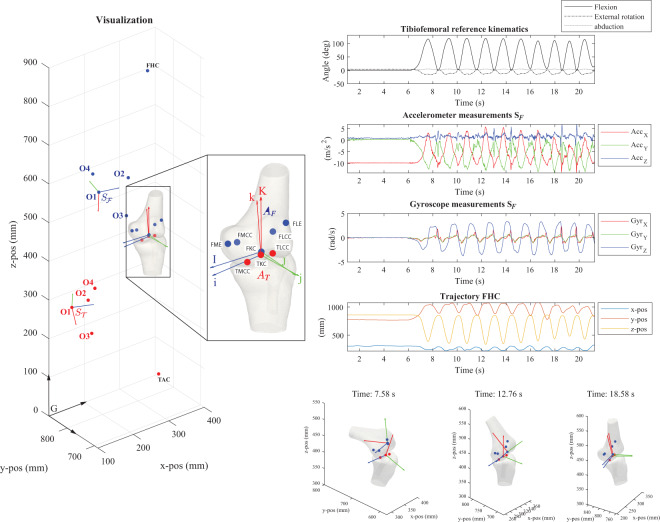
Visual and annotated representation of the multimodal data content. Reference kinematics, inertial measurements, virtual anatomical/opical marker trajectories and a representation of the relevant anatomical landmarks on the three-dimensional bone surface models (in this example: V_15_f_110.mat). Here, the specimen is in a vertical position (horizontal femoral-fixed flexion-axis). The full explanation of all abbreviations can be found in Table 1. The code for reproducing the plots for any trial is available via the public GitHub repository (https://github.com/IveW/IS2B).

All Matlab scripts for visualization and assessment of the data are provided. An example plot of the raw and processed data for one datafile is given in Fig. 3. The script to reproduce this visualization includes the transformation of coordinates starting from a global CT-scan frame *M* to a global optical motion capture frame *G*, and the identification procedure to obtain the rotations *q*^*OS*^ that align the optical marker frames *O* with the inertial frames *S*.

### Missing data

After data acquisition, we found trials of inertial sensor measurements that had a significant data-length mis-match with the optical marker trajectories. Also, we occasionally found trials with optical marker trajectories, where occlusion of the markers prevented a correct processing (with a minimum of three visible optical markers per segment). These particular datasets were dropped as annotated in the dataset summary spreadsheet^36^. Furthermore, slight deviations from the protocol as described in this study can be seen in certain trials that either started in 90° tibiofemoral flexion, instead of in a full extended pose or exceeded the desired measurement duration or RoM. All deviations of the protocol are described in the dataset summary spreadsheet. In general, the missing data does not result in any significant loss or limitation. For any combination of measurement protocol variables, there is a sufficient amount of usable data-points to infer the relation between sensor axes and anatomical axes. Additionally, depending on the IMU-based algorithm of interest, random samples from different experiments can be combined if a time-dependency is not assumed^14,33^.

## Technical Validation

The multimodal dataset of size (53 trials, 321,073 samples) is sufficient for the purpose of validating inertial-sensor-to-bone alignment strategies and inferred biomechanical parameters from inertial sensor data. The measurements of both the marker trajectories and inertial measurements needed to be temporally synchronized to be of use. The temporal synchronization was established by using a custom analog signal routed between the base stations (Lock Sync box, Vicon and Awinda Station, Xsens). A length mismatch of 2 samples (3 out of 53 trials) and one sample (24 out of 53 trials) was found. The corresponding potential time mismatch of 0.01 to 0.02 seconds should not pose a problem for the validation in most use-cases.

The raw measurement data were checked semi-automatically and manually on anomalies. We provided the constant misalignment orientations ${q}^{O{S}_{F}}$ and ${q}^{O{S}_{T}}$ for each inertial sensor and its surrounded optical cluster markers. These mis-alignment orientations were obtained from all experimental data points. To prove a rigid placement of the inertial sensor with respect to its cluster of optical markers and a correct data-match between the inertial data and optical marker trajectories, the constant misalignments were re-calculated for each trial separately. The per-file calculated misalignments deviated from the provided misalignment in the range of the expected accuracy of such sensor alignment methods^41^ with angular distances^42^ of $0.9{8}^{\circ }\pm 0.5{5}^{\circ }$ for the femur-attached inertial sensor and 0.99° ± 0.78° for the tibia-attached inertial sensor.

**Fig. 4:**
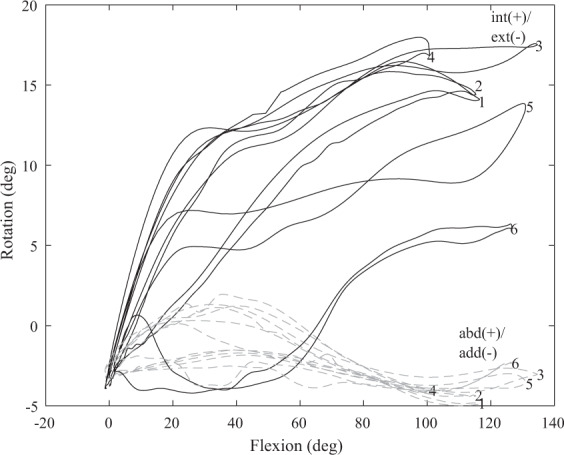
Six flexion and extending movement paths for different configurations of measurement protocol variables. To illustrate the natural coupling pattern between secondary rotations (in black: internal (int)/external (ext) rotation, in dashed gray: abduction (abd)/adduction (add)) and tibiofemoral flexion: (1) fast movement in a vertical movement plane, (2) slow movement in a vertical movement plane, (3) fast movement in a horizontal movement plane, (4) slow movement in a horizontal movement plane, (5) fast movement in a mixed movement plane, (6) slow movement in a mixed movement plane.

Unloaded motions on cadavers are often used to describe the relative movement of the bones^28,43,44^. The tibiofemoral flexion was set by the measurement protocol. It is known that secondary rotations are coupled to flexion^44^. We plotted the first flexing and extending movement path between full extension and 110 degrees tibiofemoral flexion for six trials with different configurations of measurement protocol variables in Fig. 4. The coupling pattern between secondary kinematics and tibiofemoral flexion is visible with peak internal rotations ranging up to 22.86° and abduction/abduction values ranging from 10.87° abduction to 7.62° adduction, within the expected ranges of motion^44^.

## Usage Notes

All data are available on-line in the Figshare repository^36^ and structured in the same way in Matlab compatible.mat files. Note that these files can be converted into.csv or other applicable formats for usage with other programming tools. Data are categorized in folders based on the movement plane (vertical plane, horizontal plane, mixed plane and functional movements). Additional datasets for gyroscope bias estimation, magnetometer calibration, CT-scan data and inertial sensor alignment are included in separate folders.

## Data Availability

All Matlab code used for visualization and spatial alignment is available in a public GitHub repository (https://github.com/IveW/IS2B) accompanied with detailed usage notes and commentary.
